# Beyond multivalvular disease: imaging-guided diagnosis and management of combined functional mitral and tricuspid regurgitation

**DOI:** 10.1093/ehjimp/qyaf154

**Published:** 2025-12-08

**Authors:** Prayuth Rasmeehirun, Layal Mansour, Guillaume L’Official, Marina Petersen Saadi, Erwan Donal

**Affiliations:** Inserm, LTSI—UMR 1099, University of Rennes, CHU Rennes, 2 rue Henri le Guilloux, Rennes F-35000, France; Inserm, LTSI—UMR 1099, University of Rennes, CHU Rennes, 2 rue Henri le Guilloux, Rennes F-35000, France; Inserm, LTSI—UMR 1099, University of Rennes, CHU Rennes, 2 rue Henri le Guilloux, Rennes F-35000, France; Inserm, LTSI—UMR 1099, University of Rennes, CHU Rennes, 2 rue Henri le Guilloux, Rennes F-35000, France; Inserm, LTSI—UMR 1099, University of Rennes, CHU Rennes, 2 rue Henri le Guilloux, Rennes F-35000, France

**Keywords:** mitral regurgitation, tricuspid regurgitation, echocardiography, myocardial damage, functional (secondary)

## Abstract

Combined functional mitral and tricuspid regurgitation (FMR and FTR) is now recognized not just as the coexistence of two valvular lesions, but as a distinctive clinical syndrome signalling advanced biventricular dysfunction. These lesions, although secondary to myocardial and atrial remodelling, exert a significant haemodynamic burden and perpetuate a vicious cycle of chamber dilatation, pulmonary hypertension, and symptom persistence. Medical therapy remains foundational, but many patients require sequential or combined transcatheter interventions. Optimal management requires an integrated diagnostic strategy, informed by imaging, to guide the timing and targeting of interventions for each valve.

## Graphical Abstract

**Graphical Abstract:**
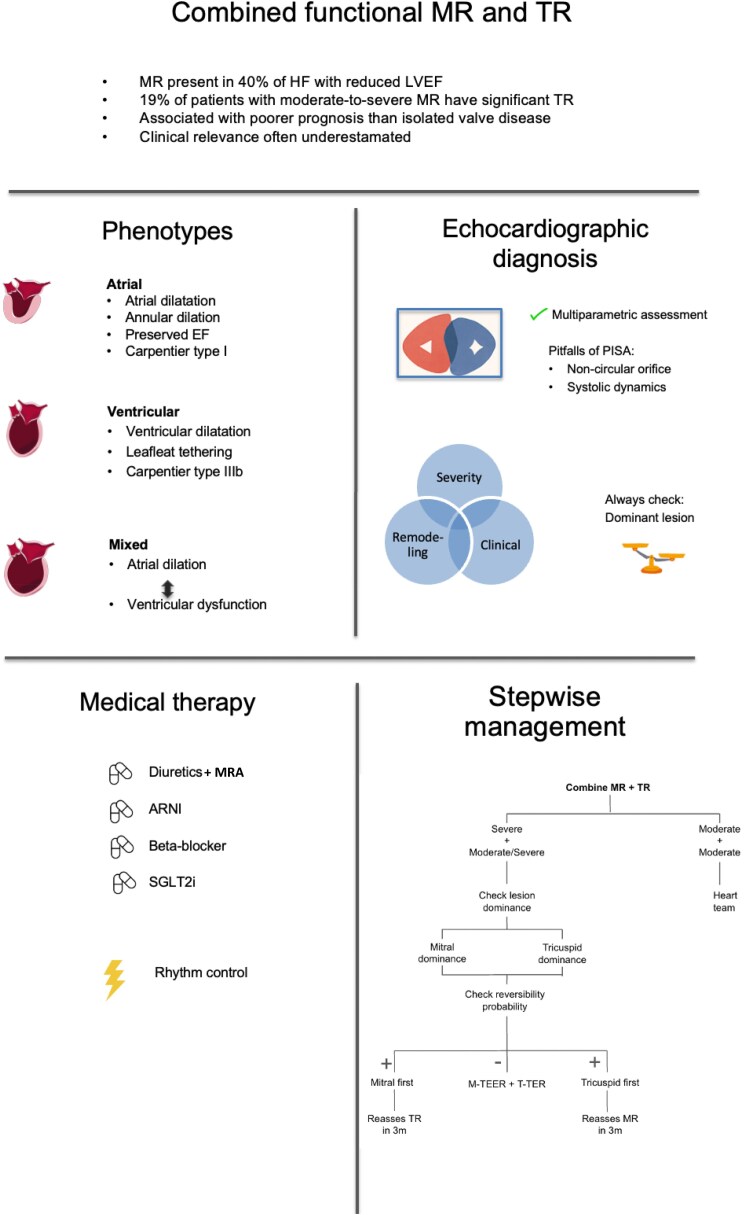
Stepwise Management Algorithm for Combined FMR and FTR. This stepwise approach supports clinical decision-making in patients with combined moderate-to-severe functional mitral and tricuspid regurgitation. Initial evaluation includes assessing the dominant lesion (mitral vs. tricuspid) and estimating the probability of reversibility of the secondary lesion. When reversibility is unlikely, or both valves are significantly diseased, simultaneous transcatheter repair (M-TEER + T-TEER) should be considered. In cases with clear dominance and potential for reverse remodelling, a staged approach is advised—starting with the dominant valve and reassessing the secondary lesion after 3 months.

## Introduction

Combined functional mitral regurgitation (FMR) and tricuspid regurgitation (FTR) are common yet often undervalued conditions, particularly prevalent among older adults with heart failure (HF) and atrial fibrillation (AF). Moderate-to-severe FMR affects up to 40% of HF patients, especially those with reduced ejection fraction (HFrEF). Significant FTR is present in approximately 19% of patients with established FMR, with prevalence increasing alongside disease progression and biventricular remodelling.^1–3^ The coexistence of FMR and FTR leads to intricate pathophysiological interactions involving left and right ventricular function, atrial dilation, pulmonary pressures, and annular dynamics, significantly influencing patient morbidity and mortality. Despite their clinical significance, combined MR and TR often receive insufficient attention in clinical practice and are typically not recognized as a distinct clinical entity, but rather as the mere coexistence of two isolated valve lesions. However, growing evidence suggests that this combination reflects a more advanced and complex disease phenotype.^4^

Patients with combined FMR and FTR exhibit higher prevalences of atrial fibrillation or flutter, pulmonary hypertension, and typically have lower left ventricular ejection fraction (LVEF), all indicative of more severe biventricular involvement and worse prognosis.^1–3^

## Mechanistic interaction between FMR and FTR

### Pathophysiology and haemodynamic interplay

FMR and FTR arise from geometric and functional disruptions of structurally normal valve leaflets due to cardiac chamber remodelling. Their coexistence reflects a bidirectional, synergistic relationship, where dysfunction in one valve can exacerbate, unmask or sustain regurgitation in the other.^5^

FMR elevates left atrial (LA) pressure and pulmonary congestion through left ventricular (LV) dilation, papillary muscle displacement, leaflet tethering, and annular dilation (Carpentier type IIIb). Elevated pulmonary pressures subsequently increase afterload on the right ventricle (RV), promoting RV dilation, dysfunction, leaflet tethering, and tricuspid annular dilation, key features of ventricular FTR. In advanced disease stages, adverse LV remodelling can impair interventricular coordination by altering septal mechanics and reducing RV contractile efficiency, leading to or worsening FTR and perpetuating biventricular dysfunction. Consequently, treating MR surgically or through transcatheter interventions may, in selected cases, significantly reduce concomitant TR.^4,6,7^

**Figure 1:**
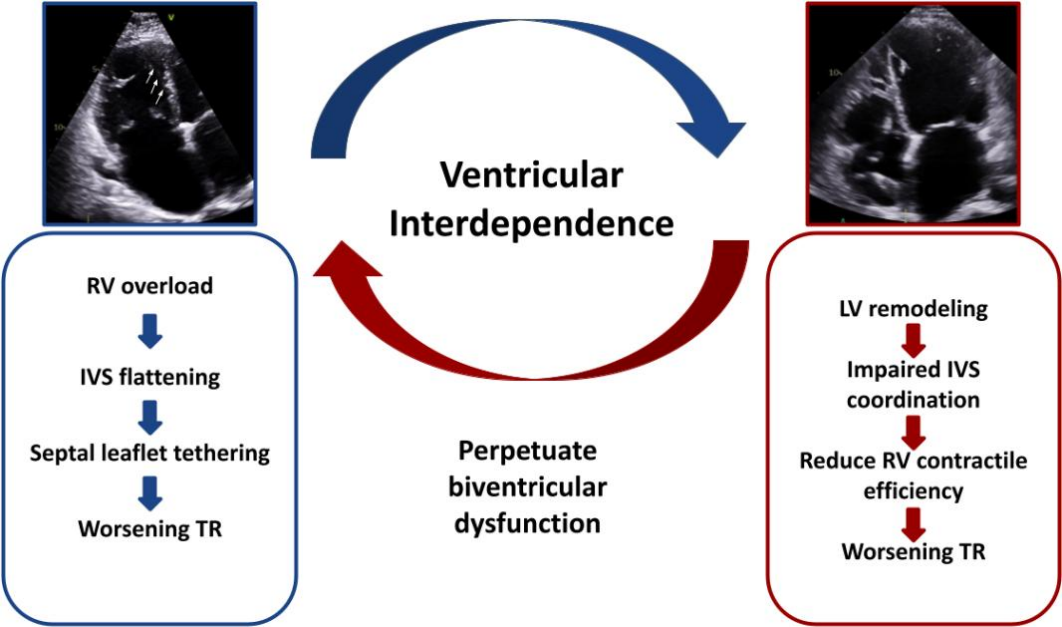
Ventricular interdependence. Right ventricular (RV) overload leads to interventricular septal (IVS) flattening and septal leaflet tethering, worsening tricuspid regurgitation. Conversely, left ventricular (LV) remodeling can impair IVS coordination and reduce RV contractile efficiency, also contributing to TR progression. This bidirectional interplay perpetuates biventricular dysfunction and highlights the importance of integrated evaluation and management.

Conversely, FTR, accounting for approximately 90% of all TR cases, adversely affects left-sided dynamics. By impairing RV output, FTR reduces LV preload, attenuates transmitral gradients, and may mask MR severity on resting imaging. Significant RV enlargement further alters LV geometry, potentially inducing dynamic MR. Beyond volumetric changes, RV dilation is often accompanied by flattening of the interventricular septum, which plays a key role in both the mechanism and reparability of TR. Septal flattening, a result of right-sided pressure overload, can be quantified using the eccentricity index and is frequently associated with restricted motion of the septal leaflet, particularly when chordae insert directly into the septum. This leads to asymmetric tethering, producing more severe and complex regurgitant jets that may be technically challenging to address. Identifying these anatomical patterns through imaging is therefore essential for procedural planning and anticipating technical complexity^8^ (*Figure 1*).

These interactions underscore a bidirectional and self-perpetuating cycle of biventricular dysfunction, highlighting the importance of simultaneous and integrated valvular evaluation and management^6,9^

This bidirectional cascade translates into more severe haemodynamic disturbances and consistently correlates with poorer clinical outcomes compared to isolated valve disease The compounded effects of biventricular dysfunction, increased filling pressures, and progressive chamber remodelling underlie this adverse prognosis.^5,10^ Residual TR after mitral valve interventions independently predicts mortality, while successful reverse remodelling post-MR correction may attenuate TR severity. These findings reinforce the necessity of an integrative, bivalvular approach in diagnostic and therapeutic strategies.

From a prognostic standpoint, the evolution from isolated atrial or ventricular phenotypes towards mixed disease represents a dynamic and progressive process. Atrial phenotypes may initially display more benign haemodynamic profiles, but chronic volume overload, sustained atrial remodelling, and pulmonary hypertension can trigger ventricular remodelling over time.

Conversely, ventricular forms may progress to involve atrial dilation and annular expansion as the disease advances. This phenotypic transition is associated with increasing regurgitant burden, RV–LV uncoupling, higher pulmonary pressures, and worsening outcomes. Recognizing this trajectory is critical for early intervention before irreversible remodelling occurs.^4,7^

### Distinction between atrial and ventricular functional MR and TR

This distinction is clinically and procedurally critical. Atrial functional regurgitation is primarily driven by atrial dilation and annular enlargement, often with preserved ventricular function, while ventricular functional regurgitation reflects adverse ventricular remodelling with leaflet tethering and more complex jet morphology. From a haemodynamic perspective, atrial phenotypes typically present with lower pulmonary pressures and preserved chamber compliance, whereas ventricular phenotypes are associated with higher filling pressures, ventricular-atrial uncoupling, and progressive remodelling. Interventional implications also differ: atrial MR/TR often responds better to annular interventions or rhythm control, while ventricular forms may require combined leaflet and ventricular-targeted strategies, with lower likelihood of spontaneous regression. Mixed forms present overlapping mechanisms and worse outcomes, highlighting the importance of early, mechanism-based phenotyping.^4,7^

FMR and FTR can be subclassified into atrial, ventricular, or mixed phenotypes, each characterized by unique anatomical, clinical, and prognostic features.

### Ventricular functional mitral regurgitation (vFMR)

vFMR typically arises from adverse left ventricular (LV) remodelling due to ischaemic or non- ischaemic cardiomyopathy, often in the setting of chronic volume or pressure overload. The underlying mechanism involves apical and lateral displacement of the papillary muscles, resulting in leaflet tethering and restricted motion, characteristics of Carpentier type IIIb dysfunction. The mitral annulus becomes flattened and dilated, with the coaptation point displaced apically. In ischaemic cases, tethering is often asymmetric and preferentially involves the posterior leaflet.^9^

From an imaging standpoint, vFMR is marked by pronounced LV dilation, increased sphericity, reduced global longitudinal strain (GLS ≤ 16%), and significantly depressed ejection fraction (often <50%). Key parameters include enlarged LV end-diastolic and end-systolic diameters, elevated LV volumes, and increased mitral leaflet tenting height and volume, as well as widened tethering angles, summarized in *Figure 4*.^11,12^ Regarding remodelling patterns, patients with vFMR usually exhibit a left atrial to LV (LA/LV) ratio ≥0.56, reflecting predominant ventricular enlargement.^13^

vFMR has been consistently associated with worse outcomes compared to atrial forms, including higher rates of all-cause mortality and heart failure hospitalization, as well as a greater likelihood of progression to mixed phenotypes.^6,12^

*Atrial Functional Mitral regurgitation (aFMR)* occurs in the context of preserved LV systolic function and is primarily driven by LA and mitral annular enlargement, typically associated with longstanding AF or heart failure with preserved ejection fraction (HFpEF). The main mechanism is isolated annular dilation (Carpentier type I): the annulus becomes enlarged but maintains a rounded geometry, and leaflet motion is preserved, without significant tethering or ventricular distortion. LA enlargement increases the anteroposterior annular dimension, impairing leaflet coaptation, while loss of atrial contractility reduces pre-systolic annular contraction, further compromising valve competence.^9^

From a remodelling perspective, patients with aFMR exhibit a LA/LV ratio > 0.56, reflecting predominant atrial enlargement. Compared to vFMR, aFMR is generally associated with more favourable outcomes, including lower rates of all-cause mortality and HF hospitalization during mid-term follow-up—likely due to less MR progression and absence of underlying cardiomyopathy.^12^

**Figure 2:**
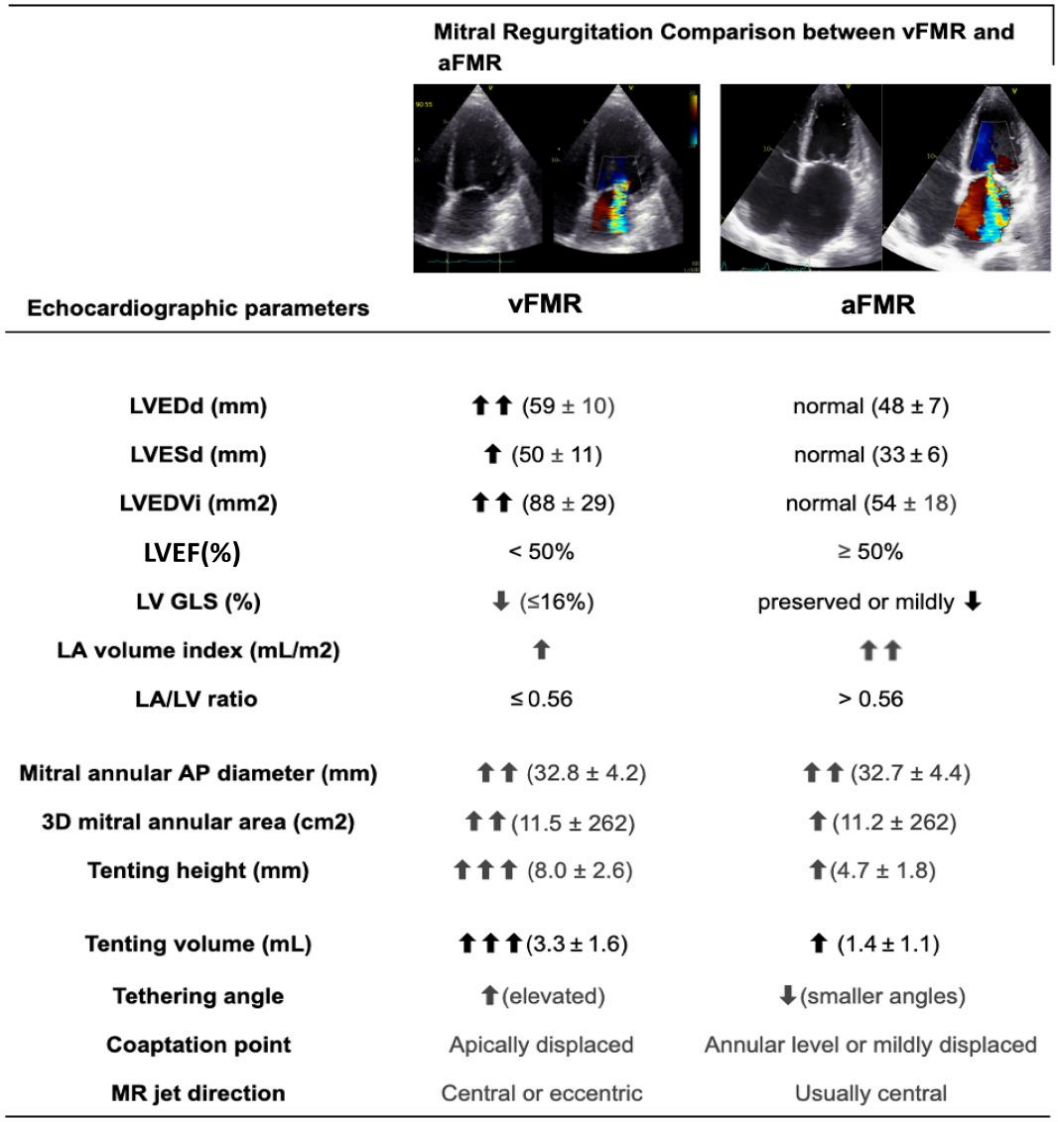
Comparative echocardiographic characteristics of ventricular vs. atrial secondary mitral regurgitation.^11^ LVEDd, left ventricular end-diastolic diameter; LVESd, left ventricular end-systolic diameter; LVEDVi, indexed left ventricular end-diastolic volume; LVEF, left ventricular ejection fraction; GLS, global longitudinal strain; LA, left atrium; AP, anteroposterior; MR, mitral regurgitation; 3D, three-dimensional.

However, recent evidence challenges the notion of aFMR as a benign condition. In a large cohort, patients with aFMR had significantly higher pulmonary artery systolic pressures and a greater prevalence of severe tricuspid regurgitation compared to those with vFMR. Additionally, among patients with severe aFMR, those with an LA/LV ratio > 0.56—indicating predominant atrial remodelling—had higher mortality than those with more pronounced LV remodelling.^13^ These findings underscore that, even with preserved LV function, atrial-driven MR can impose a substantial haemodynamic burden, contributing to right-sided remodelling and biatrial dysfunction. Key echocardiographic distinctions between aFMR and vFMR are summarized in *Figure 2*.

### Ventricular functional tricuspid regurgitation (vFTR)

**Figure 3:**
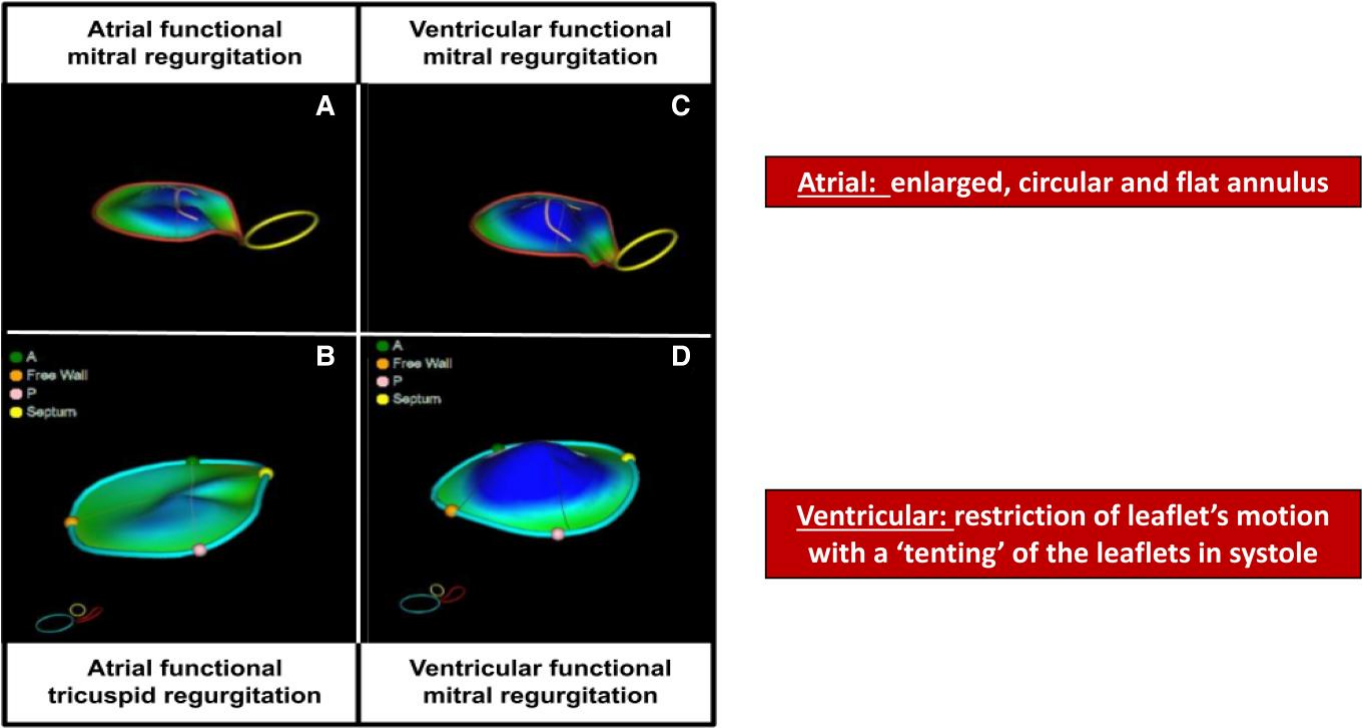
Three-dimensional annular geometry in atrial and ventricular functional regurgitations. Panels *A* and *C* illustrate 3D reconstructions of the mitral valve annulus in atrial (*A*) and ventricular (*C*) functional mitral regurgitation (FMR), while Panels *B* and *D* show the tricuspid annulus in atrial (*B*) and ventricular (*D*) functional tricuspid regurgitation (FTR). In atrial phenotypes, the annuli appear more planar and rounded, whereas in ventricular phenotypes, they are more distorted and saddle-shaped, with significant displacement of the free wall and septal components. These geometric differences highlight the distinct mechanisms underlying each phenotype and their implications for valve repair.

vFTR arises from chronic RV pressure or volume overload, most commonly due to left-sided heart disease or pulmonary hypertension. The resulting RV remodelling is characterized by dilation, spherical reshaping, and progressive systolic dysfunction. These structural changes lead to tricuspid annular enlargement, particularly along the anterior and lateral segments, and apical displacement of the papillary muscles, resulting in leaflet tethering and loss of coaptation, consistent with Carpentier type IIIb mechanism^14^ (*Figure 3*).

**Figure 4:**
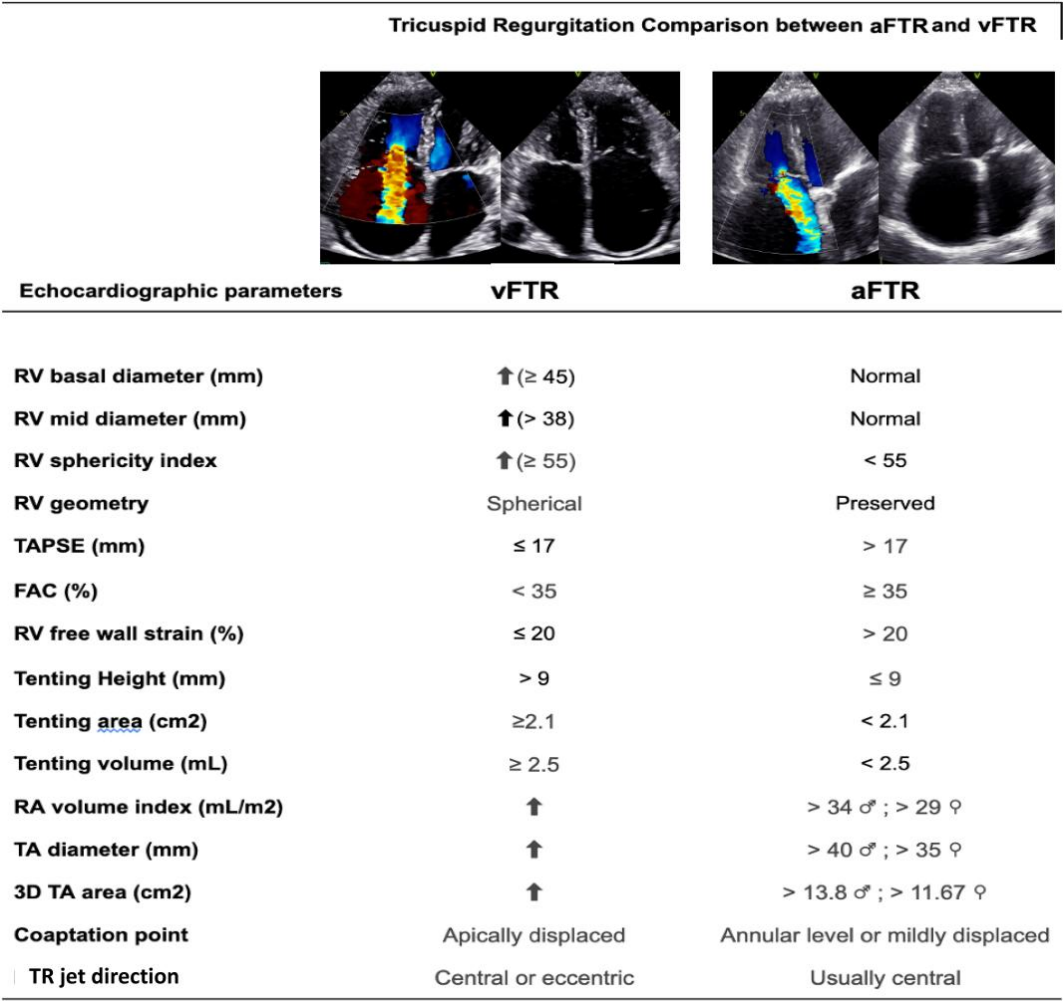
Comparative echocardiographic characteristics of ventricular vs. atrial secondary tricuspid regurgitation RV, right ventricle; RA, right atrium; LV, left ventricle; TAPSE, tricuspid annular plane systolic excursion; FAC , fractional area change; TA, tricuspid annulus; 3D, three-dimensional; ■: Men, ■: Women.

From an imaging perspective, vFTR is defined by a combination of geometric and functional abnormalities of the tricuspid apparatus and RV, as outlined in *Figure 4*. These include significant leaflet tenting, RV dilation, and impaired RV systolic performance.^7,15^

Clinically, vFTR is consistently associated with worse outcomes. Its severity correlates with increased mortality and HF hospitalizations, particularly when accompanied by RV dysfunction. In addition, residual or progressive vFTR following correction of left-sided valve lesions is a strong predictor of poor prognosis, reinforcing the importance of early identification, comprehensive biventricular assessment, and mechanism-based management strategies.^4^

### Atrial functional tricuspid regurgitation (aFTR)

aFTR arises in the context of preserved RV systolic function and is primarily driven by RA enlargement, most commonly in patients with longstanding AF. The predominant mechanism is isolated annular dilation (Carpentier type I), without significant leaflet tethering or RV geometric distortion. Annular expansion—particularly along the anterior and lateral aspects of the tricuspid annulus—compromises leaflet coaptation and leads to central regurgitant jets.^16^

Anatomically, the tricuspid valve is more susceptible to this mechanism due to its thinner fibrous annulus, broader muscular anchoring, and more compliant subvalvular apparatus compared to the mitral valve.^15,16^

Imaging-based criteria proposed by the Tricuspid Valve Academic Research Consortium (TVARC) help define the aFTR phenotype, emphasizing predominant annular and atrial remodelling in the absence of RV dysfunction or significant leaflet tethering. (*Figure 4*) Dedicated apical four-chamber views focused on the RV are essential for assessing annular geometry and leaflet coaptation (in practice, multiparametric assessment, integrating quantitative, structural, haemodynamic, and clinical markers for decision-making are key point to highlight).^4^

Although aFTR typically presents with preserved RV function, progressive RA dilation and chronic volume overload may eventually lead to RV remodelling and the emergence of a superimposed ventricular component. This progression can be accelerated by left-sided pathology such as functional MR, HFpEF, post-capillary pulmonary hypertension, or RV insults like pacemaker-induced dysfunction. As the disease advances, the distinction between atrial and ventricular phenotypes becomes less clear. Key echocardiographic differences between aFTR and vFTR are summarized in *Figure 4*.^16^

### Mixed FMR and FTR

Mixed functional regurgitation encompasses overlapping atrial and ventricular mechanisms and is frequently observed in patients with coexisting atrial fibrillation, ventricular dysfunction, or HFpEF progressing into biatrial and biventricular remodelling. In mixed MR, LA dilation and annular enlargement coexist with varying degrees of leaflet tethering and LV dysfunction.

Similarly, mixed TR often results from combined RA dilation and evolving RV remodelling with leaflet displacement.

These complex phenotypes are less responsive to isolated annular interventions and often require comprehensive, mechanism-guided strategies. Recent data from a large European cohort^17,18^ involving over 22 000 patients showed that mixed MR was the most prevalent phenotype (47%), followed by atrial (39%) and ventricular (14%) forms. Notably, mixed MR was associated with the worst clinical outcomes, including the highest rates of mortality and HF hospitalization. Additionally, patients with vFMR had a significantly greater likelihood of evolving into mixed phenotypes compared to those with atrial forms, suggesting a dynamic and progressive trajectory of disease.

Given their poor prognosis and therapeutic complexity, early recognition of mixed functional MR and TR is critical. Understanding the dominant regurgitant mechanism—atrial, ventricular, or mixed—is essential for accurate diagnosis, tailored intervention, and optimized risk stratification in patients with multivalvular functional disease.^9,12^

Severity Quantification of Functional MR and TR (practical multiparametric assessment, integrating quantitative, structural, haemodynamic, and clinical markers for decision-making). Severity grading in FMR and FTR often begins with the proximal isovelocity area (PISA) method. Common thresholds include an effective regurgitant orifice area (EROA) ≥ 30 mm² or regurgitant volume ≥60 mL for MR (though lower values may be relevant in HF or low-flow states), and EROA ≥ 40 mm² or regurgitant volume ≥ 45 mL for TR. However, PISA relies on idealized assumptions— hemispheric flow convergence and a circular, static orifice—that are rarely met in functional regurgitation, leading to frequent underestimation.^4^

**Figure 5:**
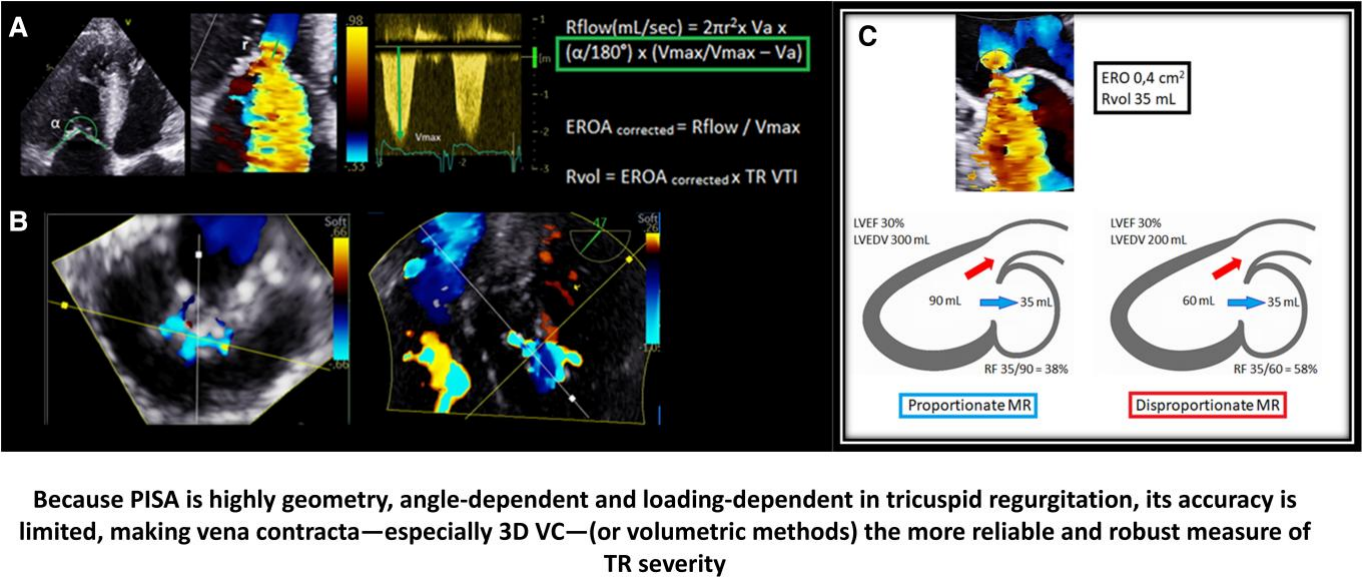
Advanced quantification of functional regurgitation: corrected PISA and 3D orifice morphology (Panel *A*): corrected PISA method for tricuspid regurgitation, using angular (α/180°) and velocity (*V*_max_/*V*_max_-*V_a_*) adjustments to account for non-hemispheric convergence and aliasing underestimation. Key measurements include flow radius (*r*), and CW Doppler *V*_max_, used to derive corrected EROA and Rvol. (Panel *B*): En face 3D colour Doppler views showing a slit-like elliptical mitral orifice (left) and an irregular, multi-lobed tricuspid orifice (right). These geometries challenge the assumptions of circular PISA and highlight the utility of 3D vena contracta area (VCA) in functional regurgitation assessment. (Panel *C*). Proportionality model for functional regurgitation. EROA, effective regurgitant orifice area.

In both FMR and FTR, the regurgitant orifice is typically elliptical, slit-like, and dynamic throughout systole. In FTR, a broad, flattened convergence zone often exceeds 180°, necessitating angle correction (α/180°) and velocity correction [*V*_max_/(*V*_max_—*V_a_*)] to improve accuracy. Additionally, low driving pressures can yield a disproportionately large EROA despite modest regurgitant volume, highlighting a potential mismatch^19,20^ (*Figure 5*).

The proportionality model reframes regurgitant severity by relating regurgitant volume to ventricular size and total stroke output. Regurgitant fraction (RF) 50% becomes a more reliable indicator, as a fixed regurgitant volume has different implications depending on chamber size. *Figure 5C* illustrates this distinction. While originally developed for FMR, the proportionality concept may also have relevance in FTR, though formal validation is pending.^21^

The same regurgitant volume has a greater haemodynamic impact in smaller ventricles. Regurgitant fraction 50% offers a more physiologic severity threshold than fixed cut-offs ( EROA, effective regurgitant orifice area; RegVol, regurgitant volume; SV, stroke volume).

Three-dimensional echocardiography, particularly 3D vena contracta area (VCA) planimetry, overcomes 2D PISA’s geometric limitations by allowing en face visualization of irregular regurgitant orifices. Guideline-endorsed TR thresholds for 3D VCA are ≥75 mm² (severe), ≥95 mm² (massive), and ≥115 mm² (torrential). For MR, a threshold >0.4 cm² has been proposed, though outcome validation is still evolving.^4,7,22^

Overall, while 2D PISA remains widely used, its limitations—particularly in functional and multivalvular disease—underscore the need for a multiparametric approach. Incorporating chamber size, flow dynamics, and 3D imaging improves accuracy and strengthens the foundation for procedural decision-making.

For the quantification of the regurgitation: For functional mitral and tricuspid regurgitation, vena contracta—and ideally 3D vena contracta—is the reference quantitative technique, providing geometry-independent assessment that overcomes the intrinsic assumptions and limitations of the PISA method.^22^

Emerging quantitative methods are reshaping the assessment of MR and TR severity by providing geometry-independent, reproducible measurements. Three-dimensional vena contracta, automated 3D quantification, and AI-enhanced flow analysis offer more robust evaluation than traditional PISA-based approaches. These tools better capture complex regurgitant orifice morphology and dynamic valve behaviour, improving clinical decision-making and patient selection for transcatheter intervention.^22,23,24^

The TVARC criteria provide a standardized, clinically meaningful framework for grading tricuspid regurgitation severity, integrating both quantitative and qualitative parameters. They help capture the true spectrum of TR, particularly at the severe end, and support consistent patient selection and outcome assessment in transcatheter therapies.^7^

*Diagnostic phenotyping and lesion dominance in combined FMR and FTR*—Structured Stepwise Clinical Pathway (practical multiparametric assessment: integrating quantitative, structural, haemodynamic, and clinical markers for decision-making).

In patients with combined FMR and FTR, identifying the dominant lesion is critical for therapeutic planning. Lesion dominance refers to the valve contributing most significantly to remodelling, haemodynamic burden, and symptoms—shifting the focus from numeric grading to identifying the primary driver of dysfunction.^4^

A practical approach integrates three domains: (i) quantitative severity, (ii) structural remodelling, and (iii) clinical presentation. Concordance across these areas supports a confident diagnosis. Quantitative markers include EROA, regurgitant volume, VCA, and venous flow patterns (systolic reversal in pulmonary or hepatic veins). Structural indicators such as LA or LV-dilation and mitral leaflet tenting suggest MR dominance, while RA or RV enlargement and tricuspid leaflet tethering favour TR. Symptomatically, MR is associated with dyspnoea and pulmonary congestion, whereas TR more often presents with oedema, ascites, and jugular venous distention. These phenotypic contrasts are summarized in *Tables 1* and *2*.

**Table 1:** Diagnostic features used to determine lesion dominance in combined functional MR and TR

Domain	Marker/Feature	MR dominance	TR dominance
Quantitative Severity	EROA, Regurgitant Volume, VCA, Flow Reversal	Pulmonary vein systolic reversal, large EROA	Hepatic vein systolic reversal, large EROA
Structural Remodelling	Chamber and valve geometry	LA or LV enlargement,	RA or RV enlargement, tricuspid tethering
Clinical Presentation	Symptom profile	Dyspnea, orthopnoea, pulmonary oedema	Oedema, ascites, jugular venous distention

MR, mitral regurgitation; TR, tricuspid regurgitation; EROA, effective regurgitant orifice area; VCA, vena contracta area; LA, left atrium; LV, left ventricle; RA, right atrium; RV, right ventricle.

**Table 2:** Prediction of transcatheter edge-to-edge repair of the tricuspid regurgitation^6,25,28,45,48^

Predictor category	Specific parameters
Right atrial remodelling	• RA volume (baseline and reverse remodeling)
Tricuspid annulus	• Annular diameter (2D/3D) • Annular area and dynamics
TR Phenotype	• Atrial vs. Ventricular TR phenotype • Presence of PM-induced TR
Atrial fibrillation	• Presence of AF • Duration and chronicity of AF
RV Function	• TAPSE, RVFAC • RV free-wall strain • RV-PA coupling markers (TAPSE/sPAP)
Leaflet tethering/coaptation	• Tethering height • Tethering area • Coaptation gap
Loading conditions	• SPAP/pulmonary pressures • Volume status
Procedural TR response	• Immediate TR reduction post-M-TEER • Improvement in LA pressure/V-wave
Leaflet tethering/coaptation	• Tethering height • Tethering area • Coaptation gap

When all domains align, the dominant lesion is clearly defined. However, overlap is common. Both regurgitations may reach severity thresholds or display shared remodelling, particularly in patients with biatrial dilation, biventricular dysfunction, or longstanding AF. These co-dominant phenotypes may require individualized or staged treatment. When dominance remains uncertain, greater weight is typically placed on the valve more clearly linked to structural remodelling and symptomatic burden.

**Figure 6:**
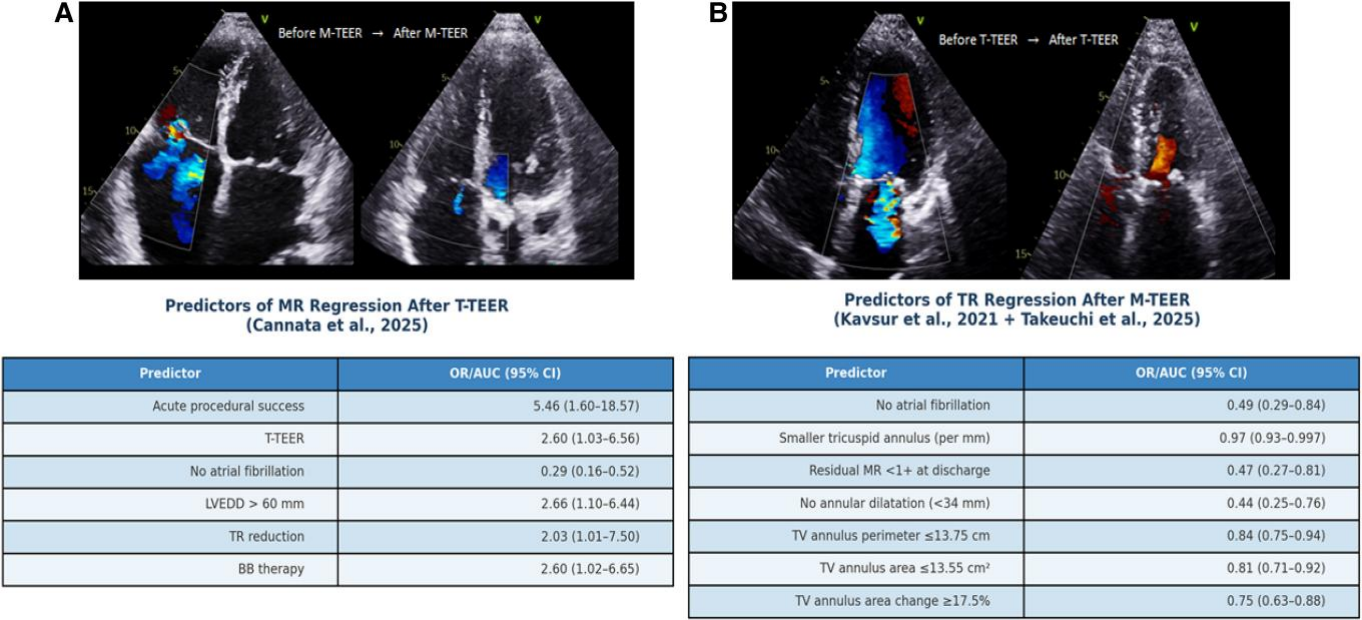
Echocardiographic improvement and independent predictors of mitral and tricuspid regurgitation regression following TEER. Top: colour Doppler images before and after M-TEER (left) and T-TEER (right). Bottom: predictors of regurgitation regression from multivariable models.^25,26,27^ All predictors shown were statistically significant (*P* < 0.05). OR, odds ratio; AUC, area under the curve; CI, confidence interval.

Importantly, the secondary lesion is not a benign bystander. Persistent TR after M-TEER, or residual MR after T-TEER, has been consistently associated with worse outcomes, even when the primary procedure is technically successful.^17,25^ Prognosis may therefore depend not only on correcting the dominant lesion but also on anticipating the behaviour of the secondary one. Regression is more likely when the secondary lesion is mild, annular dilation is limited, tethering is absent, and AF is recent or paroxysmal. Atrial-type TR and smaller RA volumes, for example, favour improvement after mitral repair.^26,27^ Conversely, severe annular enlargement, fixed leaflet restriction, or longstanding remodelling—particularly in patients with permanent atrial fibrillation—predict persistence. In such cases, preprocedural recognition is essential to determine whether a combined or staged intervention is needed (*Figure 6*).

### Clinical, prognostic, and anatomical feasibility assessment of clinical indication (multiparametric assessment)

Transcatheter intervention for combined FMR and FTR hinges on identifying the dominant regurgitant lesion. Following the 2025 ESC/EACTS framework for isolated valve pathology, treatment should only be considered when the dominant lesion causes persistent symptoms despite optimized guideline-directed medical therapy (GDMT) and is confirmed as severe by comprehensive imaging. Severity assessment must account for volume status, heart rate, and rhythm—especially in AF—given their influence on regurgitant measurements.^28^

The non-dominant valve is not usually targeted upfront. If it is mild with a low likelihood of persistence, conservative monitoring suffices. However, if moderate or severe with anticipated persistence, simultaneous or staged treatment may be warranted, contingent on anatomical feasibility. The ‘mod–mod’ phenotype (combined moderate MR and TR) is increasingly linked to adverse outcomes, particularly in patients with atrial remodelling or heart failure with preserved ejection fraction (HFpEF),^29^ necessitating individualized management despite not being formally actionable by current guidelines.

### Prognostic stratification and functional reserve

Even when clinical criteria are met, transcatheter intervention should proceed only if sufficient cardiac reserve exists. Prognostic assessment integrates LV and RV function, atrial remodelling, and pulmonary pressures, interpreted in the context of lesion dominance.

For dominant MR, key prognostic parameters include the following:

1. Left ventricular global longitudinal strain (GLS) > −7%, reflecting impaired LV contractile reserve and predicting limited symptomatic or survival benefit after M-TEER.^30^
2. Right ventricular free-wall strain ≥ −18%, indicating early RV dysfunction, particularly in the presence of pulmonary hypertension.
3. TAPSE/sPAP ratio < 0.36 mm/mmHg, suggesting RV–pulmonary artery uncoupling and reduced event- free survival.^31,32^

For dominant TR, two right-sided markers are crucial:

1. TAPSE/sPAP ratio ≤ 0.406 mm/mmHg, strongly associated with one-year mortality and impaired RV reserve.^33^
2. Right ventricular fractional area change (RVFAC) < 35%, indicating significant RV systolic dysfunction and worse outcomes unless function improves post- intervention.

These thresholds help identify patients most likely to benefit from intervention. As evidence evolves, additional markers may further refine selection.^34^

### Anatomical feasibility

Anatomical screening is the final determinant for transcatheter treatment, with distinct feasibility criteria for repair vs. replacement strategies.

For FMR, M-TEER is generally preferred given its broader anatomical applicability. It requires leaflet length ≤ 7 mm, a tenting height < 10 mm, coaptation length > 2 mm, and a mitral valve area >3.0 cm². Excessive leaflet tethering or annular calcification may reduce procedural success. Transcatheter mitral valve replacement (TMVR) bypasses leaflet limitations but demands a larger annular area (≥8 cm²), sufficient neo-left ventricular outflow tract (LVOT) area (≥1.7–1.9cm²), and may be contraindicated in cases of severe subvalvular distortion.^35,36^

For FTR, T-TEER is most effective when the coaptation gap in front of the ‘grasping zone’ is <7 mm, the regurgitant jet is central or anteroseptal, and the valve is tri-leaflet with preserved mobility. Feasibility declines with severe annular dilation, leaflet tethering, or spherical RV geometry. In such anatomies, Transcatheter tricuspid valve replacement (TTVR) may be considered, though it requires suitable anchoring anatomy and is limited by device-specific annular size thresholds (typically <40–70 mm).^37,38^ Cardiac implanted electronic devices (CIED) lead interference, RA/RV dilation, and annular calcification must also be assessed, particularly when device stability or delivery may be compromised.^39^

Ultimately, repair is favoured when leaflet geometry permits, but replacement offers a more definitive solution in anatomically unsuitable cases—albeit with stricter screening criteria and higher exclusion rates. Imaging-based triage remains central to procedural planning and device selection.

### Treatment strategy: medical optimization and intervention planning (multiparametric assessment: integrating quantitative, structural, haemodynamic, and clinical markers for decision-making).

#### Medical optimization and procedural synergy

GDMT remains essential in FMR and FTR, particularly in high-risk or comorbid patients. Its aims are to relieve congestion, modulate neurohormonal pathways (renin–angiotensin–aldosterone system, sympathetic nervous system), and manage atrial fibrillation. Loop diuretics are first-line; in resistant cases, adding a thiazide-type agent can enhance natriuresis via sequential nephron blockade.^40^ Mineralocorticoid receptor antagonists offer both natriuretic and antifibrotic benefit—Structured Stepwise Clinical Pathway.

For HFrEF, beta-blockers, angiotensin receptor–neprilysin inhibitors (ARNIs), mineralocorticoid receptor antagonists (MRA), and SGLT2 inhibitors are the core therapeutic agents. ARNIs are preferred over angiotensine converting enzyme inhibitors or angiotensin II receptors blockers when tolerated, due to superior survival and hospitalization outcomes. SGLT2 inhibitors also lower preload and afterload with minimal hypotension. Inotropes may support low-output states or cardiogenic shock but do not modify the disease. In atrial functional regurgitation, rhythm control may aid remodelling and coaptation, but is rarely sufficient alone.^41–43^

GDMT seldom reverses significant regurgitation once remodelling is established. Its value lies increasingly in procedural facilitation: by optimizing load, it may reduce annular dimensions or coaptation gap, improving repair feasibility—especially in staged approaches. Conversely, successful M-TEER may improve GDMT tolerance by restoring forward output and relieving pulmonary congestion. This reciprocal interaction—where GDMT enhances procedural candidacy, and intervention enables medical optimization—supports a flexible, imaging-guided treatment strategy.^44^

Recent data from the TriValve registry, the TRI.FR randomized trial, and Cannata *et al.*^25,45^ have refined our understanding of procedural sequencing and TR regression after MR repair. These studies highlight that TR regression is more likely in patients with atrial TR phenotype, limited annular dilation, preserved RV function, and absence of advanced AF. Matsumoto *et al.*^17^ further confirmed that early MR correction with M-TEER can reduce functional TR in selected patients, with maximal benefit observed within 3 months. However, persistent TR—particularly ventricular or mixed types—is strongly associated with adverse outcomes, underscoring the importance of early phenotyping and stratification.

Updated analyses comparing ‘mitral-first’ vs. ‘tricuspid-first’ strategies suggest that an MR-first approach is preferable in most patients, as untreated MR may worsen after isolated TR repair in up to 11% of cases. Predictors of TR regression after MR repair include smaller annular perimeter, lower RA volume, absence of AF, and less tethering.^25,27^ Conversely, severe annular dilation, longstanding AF, and RV dysfunction identify patients unlikely to regress and may benefit from simultaneous or staged T-TEER. These findings support an individualized, mechanism-driven sequencing strategy.

#### Strategic valve selection and treatment sequencing

Following GDMT optimization, the next decision is whether to intervene on one valve or both. In some patients, correcting the dominant lesion may lead to regression of the secondary lesion or improve anatomical conditions for a second-stage repair. In others, both valves require treatment, either sequentially or during the same procedure.

When MR is dominant, M-TEER is typically performed first. TR may regress following MR correction, especially in phenotypes without advanced AF, significant annular dilation, or RV dysfunction. If regression is anticipated, reassessment should occur at 3 months, further improvement beyond this point is unlikely, and persistent lesions—particularly TR after M-TEER—have been independently associated with early mortality. Even when TR persists, prior MR correction may facilitate tricuspid repair by reducing annular dimensions or coaptation gap.^25,26,46^

Conversely, a tricuspid-first strategy may be considered in isolated atrial TR or advanced right-sided remodelling. However, this approach carries a risk of MR worsening in up to 11% of patients, possibly due to septal shift or altered mitral geometry impairing leaflet coaptation. This can lead to pulmonary congestion and clinical deterioration. Even in cases with severe TR and only moderate MR, an MR-first strategy may be safer—unless MR is clearly load-driven and expected to regress after TR repair.

In patients with severe bivalvular disease and predictors of non-regression, both valves may require treatment. This can be achieved either concurrently or in stages. Although combined repair has shown acceptable periprocedural safety in experienced centres, long-term data on durability and functional improvement remain limited. By contrast, a staged approach leverages the iterative nature of transcatheter therapy—allowing each valve to be addressed at the optimal anatomical and physiological moment, guided by real-time imaging and evolving response. Mitral-first is generally favoured in this strategy, given the higher haemodynamic risk of MR worsening compared to residual TR.^47^

According to current ESC/EACTS guidelines, surgical management of tricuspid regurgitation should be systematically integrated into the treatment of patients undergoing mitral valve surgery. Concomitant tricuspid valve repair is *recommended* in the presence of severe TR, and *should be considered* even for moderate TR or for mild TR with significant annular dilatation, as isolated correction of the mitral lesion rarely leads to improvement of TR and late reoperation carries high risk. This combined approach prevents progression of TR, limits adverse right-sided remodelling, and aligns with evidence showing better long-term structural and clinical outcomes^28^

#### Structured stepwise clinical pathway

Transcatheter therapy thus offers a unique advantage: it supports a flexible, stepwise strategy that integrates procedural feasibility with disease trajectory, making individualization the cornerstone of modern structural intervention (*Graphical Abstract*).

*Five major evidence gaps continue to limit the management of patients with combined MR and TR:*

1. There are currently no adequately powered randomized trials evaluating outcomes of transcatheter or surgical dual-valve interventions. Most existing data are extrapolated from isolated MR or TR trials, which limits the generalizability of procedural sequencing strategies.
2. Major landmark trials systematically excluded patients with more than mild secondary lesions in the contralateral valve, resulting in a profound lack of evidence for this large and high-risk subgroup.
3. Current guidelines address MR and TR independently.^28^ They offer no integrated framework for transcatheter bivalvular interventions and do not provide timing or sequencing recommendations beyond surgical concomitant repair.^28^
4. Existing risk stratification models do not incorporate biventricular interdependence or dynamic lesion interactions. This gap highlights the need for integrated imaging and AI-driven predictive tools to refine patient selection and procedural strategy.
5. Most published studies focus on early mortality and rehospitalization, with scarce data on functional capacity,^4,7,28^ quality of life, reverse remodelling, and durability of dual-valve repair or replacement. Understanding these trajectories is critical for defining optimal procedural timing.

Together, these gaps represent key opportunities for future research: conducting pragmatic randomized trials, integrating dual-valve patients into registries, refining guideline recommendations, and developing validated, physiology-guided procedural algorithms supported by advanced imaging and AI prediction models.

### Future directions: the next step is knowing when

Treating combined FMR and FTR is not a technical problem—it is a strategic one. We are no longer limited by access or devices. We are limited by timing. We delay intervention when patients appear stable on medical therapy, only to act when remodelling is irreversible or rhythm is lost. The field does not need more procedural enthusiasm—it needs clarity on when to act, who will benefit, and how to adapt as device options expand. Three priorities stand out.

### Predicting progression or regression—before it happens

Our current strategy assumes that one valve is dominant and the other might improve. Sometimes that is true. Often, it is not. We need predictive models—driven by AI and built from imaging, rhythm burden, and chamber strain—that tell us what will happen after the first valve is treated. A regression score or progression risk model would change everything: timing, staging, and even the patient's understanding of their disease. Until then, we are guessing.

### Imaging criteria for combined disease—not two separate grades

Combined MR and TR is not just ‘moderate plus moderate.’ It is a unique volume overload state with overlapping remodelling. We need imaging frameworks that account for total regurgitant burden, atrial compliance, RV compensation, and how these change under stress. A bivalvular staging system—rooted in physiology, not just anatomy—would finally give clinicians a coherent basis to act before irreversible damage occurs.

### The devices are coming—and will outpace our strategy

Combined leaflet repair, annuloplasty plus clip, staged replacement, dual-valve platforms— they are all arriving. And they will make clinical decisions harder, not easier. Do we fix both at once or stage them? Clip one and replace the other? Without integrated decision pathways, more tools will just mean more confusion. Strategy must evolve alongside devices—or we risk choosing based on availability, not biology.

## Conclusion

**In conclusion,** the management of combined functional mitral and tricuspid regurgitation must be strategic, physiology-guided, and individualized. Rather than viewing MR and TR as isolated entities, clinicians should embrace an integrated, bivalvular strategy anchored in precise phenotyping, multimodality imaging, and haemodynamic assessment. Future care pathways must incorporate AI-driven predictive models to anticipate lesion progression, determine the likelihood of TR regression after MR repair, and support real-time procedural decision-making.

Equally critical is the establishment of evidence-based procedural sequencing frameworks. Building on recent, MR-first remains appropriate in most patients, but selection should be guided by phenotype, annular geometry, and RV function. Predictors of non-regression should prompt early combined or staged treatment. Integrating these physiological principles will allow earlier, targeted interventions to prevent irreversible remodelling and improve long-term outcomes.

As device technologies evolve, the challenge is no longer ‘can we treat both valves’ but ‘when and in what sequence.’ This shift demands structured decision algorithms, imaging-led triage, and AI-enhanced prediction tools that transform procedural planning from empirical to evidence-driven practice.

## Data Availability

The data underlying this article will be shared on reasonable request to the corresponding author.

## Lead author biography

Erwan DONAL, MD, PhD, FESC, is leading the imaging lab, the cardiomyopathy referral center, and the cardiac echo core lab in CHU Rennes (erwan.donal@chu-rennes.fr).
